# Magnetic resonance imaging findings of idiopathic granulomatous mastitis: can it be an indirect sign of treatment success or fail?

**DOI:** 10.1186/s12880-019-0397-2

**Published:** 2019-12-16

**Authors:** Ayşegül Altunkeser, Fatma Zeynep Arslan, Mehmet Ali Eryılmaz

**Affiliations:** 1 Department of Radiology, University of Health Science, Konya Trainning and Research Hospital, Konya, Turkey; 2 Department of General Surgery, University of Health Science, Konya Trainning and Research Hospital, Konya, Turkey

Idiopathic granulomatous mastitis (IGM) is a rare idiopathic benign inflammatory disease of the breast that is characterized by the formation of non-necrotizing granulomas and sterile micro-abscesses [1]. Although several treatment options are available for IGM, there is no well-established treatment algorithm for the disease [2]. Antibiotics, surgical drainage, a partial mastectomy, steroids and methotrexate are among the treatment modalities. Although good results have been achieved with the available modalities in some patients, treatment success has not been achieved in a large number of patients, who then require a mastectomy. To date, only a few studies have investigated magnetic resonance imaging (MRI) findings of IGM [3–5]. According to the literature, morphological and contrast-enhancement features on MRI in cases of IGM show great variety, depending on the IGM stages [e.g. inflammatory reactions, abscesses and fibrosis [4, 5]. To the best of our knowledge, no previous studies have investigated the potential importance of MRI findings in predicting treatment success. The aim of the present study was to determine whether MRI findings could play a role in predicting treatment success or guiding the choice of treatment.

## Material and methods

This retrospective study was conducted in accordance with the ethical guidelines of the Declaration of Helsinki and was approved by the local committee of our hospital. Sixty consecutive female patients who were diagnosed with IGM between 2010 and 2018 were included in the study.

The clinical and pathological findings were obtained from a local database in the hospital. All the patients were diagnosed with IGM in our hospital and treated in the general surgery department of our hospital. A core needle biopsy was taken, and the diagnosis of IGM was histopathologically confirmed. Grocott, Periodic acid–Schiff (PAS) and Ziehl–Neelsen staining were negative in all the patients. Patients with other types of granulomatous mastitis [sarcoidosis, tuberculosis etc.] were excluded from the study.

All the patients were first evaluated by a general surgeon. After a physical examination and anamnesis, they were referred for sonographic and mammographic evaluations. All of our patients underwent an MRI scan before treatment. A radiologist (AA) experienced in the field of breast imaging performed all the assessments. The lesions were categorized in the light of breast imaging reporting and data system (BI-RADS) lexicon established by the American college of radiology [6]. The involvement of IGM in was grouped as retroareolar region or other quadrant. Mass lesions and non-mass enhancements (NMEs) were noted. Subsequently, morphological features of the mass lesions were identified based on their shapes (irregular, round or oval), margins (irregular, well circumscribed or speculated) and internal enhancement features (heterogeneous, homogeneous or rim). The NMEs were classified depending on their distribution (regional, diffuse, focal, linear, multiple regional or segmental) and internal enhancement patterns (heterogeneous, homogeneous, clumped or clustered). The presence of a fistula clinically detected and also seen on MRI was recorded. The treatment methods were categorized as medical [corticosteroids or non-steroidal anti-inflammatory agents], surgical [segmental mastectomy] and drainage. Antibiotic and nonsteroidal antiinflammatory agents were the basis of our medical treatment. The corticosteroid treatment consisted of prednisolone, which was administered initially at a dose of 60 mg daily in divided doses via the oral route. The doses were tapered slowly, depending on clinical improvements. The asymptomatic cases and BI-RADS 1 and 2 on radiological follow-ups were accepted as clinical and radiological improvement, and those were evaluated as successful treatment. The relationship between the MRI findings, presence of fistula formation and treatment methods with treatment success was investigated.

### MRI protocol

All the examinations were performed using a dedicated 16-channel double breast coil, with 1.5 Tesla MRI (Magnetom Aera; Siemens Healthcare, Erlangen, Germany) equipped with 45-mT m gradients. The patient was placed in the prone position on a table. The imaging protocol was bilateral. The coronal flashed-grappa (TR/TE: 417/11 ms, matrix: 352 × 384, slice thickness: 3 mm, FOV: 180–500 mm), (matrix:352 × 384, slice thickness: 3 mm, FOV: 280–300 mm) T1W started with sequences. Then, the T2W TIRM (TR/TE: 2770/66 ms, matrix: 352 × 384, inversion time: 150 ms, flip angle: 150 degrees, spatial resolution: 0.7 × 0.7 × 2 mm, acquisition time: 3 min 26 s) DWI (TR/TE: 6200/88, long distance resolution: 2.7 × 2.7 × 4 mm, slice thickness: 3 mm, FOV: 258–300 mm, B values 0 and 800 s/mm2, spectral fat saturation and acquisition time: min 47 s). A dynamic study was performed using FLASH (TR/TE: 4.79/1.70 msec, spatial resolution: 0.8 × 0.8 × 1.3 mm, cross-sectional thickness: 1.6 mm, FOV: 318–500 mm). Gadopentetate dimeglumine was intravenously administered at a dose of 0.1 mmol/kg of body weight.

### Statistical analysis

The statistical analysis was performed using SPSS 22 software [IBM Corporation]. The results of the descriptive statistics are presented as mean ± standard deviation [SD] and min-max. The relationship between MRI findings and treatment success was investigated using a chi-square test and univariate logistic regression. *P* value of < 0.05 was considered statistically significant.

## Results

**Fig. 1:**
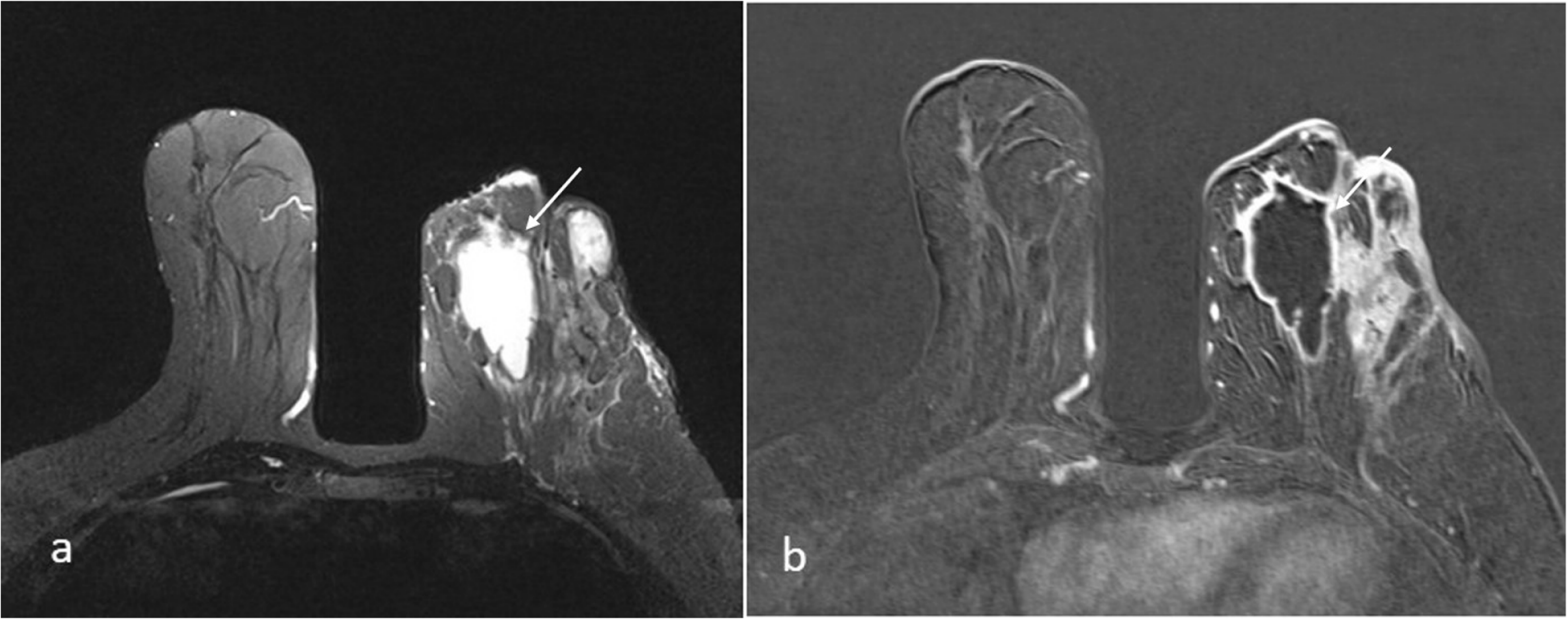
On **a** T2W TIRM image, **b** Contrast enhanced subtraction image of 34 year-old patients; multiple abcess formations (arrow), rim-like enhancement and diffuse edeame is seen in the left breast

In total, 60 IGM patients [62 breast lesions] were re-evaluated retrospectively. The median age of the patients was 35 y (range: 23–65). All the patients were followed up for between 1 and 7 y, with a mean follow up time of 2.32 y. In the study population, 35 of the patients suffered from mastodinia, and rest of them complained from hardness. Two patients were bilaterally affected (Table 1). Seven patients had retroareolar involvement. In these patients, the treatment failed in two cases, and IGM recurred in one patient. In patients with retroareolar regional involvement, treatment success was significantly lower than that of the patients with IGM involving the other quadrants (*p* < 0.05). MRI revealed a mass lesion in 3 patients, NME in 15 patients and both a mass lesion and NME in 44 patients. Regarding the MRI features of the mass lesions, in 28 cases, the mass was round shaped. In 33 cases, the mass was well circumscribed. The mass enhancement was mostly of the rim type in 41 patients. Abscesses were detected in 39 patients (Fig. 1). In 33 patients, the NME was regional, and the internal enhancement pattern was mostly heterogeneous (*n* = 31). Thirty six of the lesions were multifocal, 16 were multicentric and 6 were focal based on the findings of non-mass enhancement. There was no statistically significant association between the MRI findings and treatment success (Tables 2 and Table 3).

**Table 1:** General descriptive features

Parameters	*n*	*Mean ± SD*	*Min-Max*
Age	60	35.2 ± 8.73	23–65
Mean follow up time	60	2.32 ± 1.47	1–7
	*n*^a^	*%*	
Complaint			
Mastodinia	35	56.5	
Hardness	27	43.5	
Side			
Single breast	58	96.7	
Bilateral involvement	2	3.3	
Breast quadrant			
Other quadrants	55	88.7	
Retroareolar	7	11.3	

*n* Number of patients, *n*^a^ Number of lesions

**Table 2:** The relationship between MRI findings and the treatment success

Parameters	Treatment success				
	Recurrence (%)	Yes (%)	No (%)	Total	*p*
Quadrants					
Other quadrants	0 (%0.0)	40 (%90.9)	15 (%88.2)	55	0.018*
Retroareolar	1 (%100)	4 (%9.1)	2 (%11.8)	7	
MRG lesion					
Mass	0 (%0.0)	2 (%4.5)	1 (%5.9)	3	0.976
Mass + NME	1 (%100)	31 (%70.5)	12 (%70.6)	44	
NME	0 (%0)	11 (%25.0)	4 (%23.5)	15	
MRI mass					
Abcess	1 (%100)	27 (%81.8)	11 (%84.6)	39	0.878
Solid	0 (%0)	6 (%18.2)	2 (%15.4)	8	
Shape					
Irregular	0 (%0)	12 (%36.4)	4 (%30.8)	16	0.544
Oval	0 (%0)	1 (%3)	2 (%15.4)	3	
Round	1 (%100)	20 (%60.6)	7 (%53.8)	28	
Margin					
Irregular	0 (%0)	9 (%27.3)	3 (%23.1)	12	0.914
Well-circumsribed	1 (%100)	23 (%69.7)	9 (%69.2)	33	
Spiculated	0 (%0)	1 (%3)	1 (%7.7)	2	
Mass enhancement					
Heterogeneous	0 (%0)	4 (%12.1)	1 (%7.7)	5	0.570
Homogeneous	0 (%0)	0 (%0)	1 (%7.7)	1	
Rim enhancement	1 (%100)	29 (%87.9)	11 (%84.6)	41	
Nonmass enhancement					
Regional	0 (%0)	22 (%53.7)	11 (%68.8)	33	0.271
Diffusse	0 (%0)	2 (%4.9)	1 (%6.3)	3	
Focal	1 (%100)	5 (%12.2)	0 (%0)	6	
Lineer	0 (%0)	2 (%4.9)	0 (%0)	2	
Multiple regional	0 (%0)	9 (%22)	4 (%25)	13	
Segmental	0 (%0)	1 (%)	0 (%0)	1	
NME (internal)					
Heterogeneous	0 (%0)	25 (%59.5)	6 (%37.5)	31	0.298
Homogeneous	0 (%0)	8 (%19)	6 (%37.5)	14	
Clumped	0 (%0)	2 (%4.8)	1 (%6.3)	3	
Clustered	1 (%100)	7 (%16.7)	3 (%18.8)	11	

*P* value less than 0.05 was considered as statistically significant. *: statistically significant. *NME* Nonmass enhancement

**Table 3:** The association between MRI findings and treatment success

Variables	Treatment Success (Univariate Logistic Regression)		
	Odds Ratio	95% CI	*p* value
Breast Mass	0.866	0.363 to 2.066	0.746
Shape	1.806	0.587 to 5.559	0.303
Margin	0.927	0.305 to 2.815	0.893
Mass enhancement	1.657	0.399 to 6.876	0.487
NME (internal)	1.014	0.576 to 1.784	0.961
Treatment Methods	0.670	0.373 to 1.205	0.181
Fistula Formation	0.765	0.241 to 2.427	0.649

*P value less than 0.05 was considered as statistically significant*

*NME* Nonmass enhancement

Medical treatment was successful in 18 patients, IGM resolved in 20 patients with drainage, and surgery was successful in 6 patients. IGM recurred in one patient who underwent drainage. Fistulas were observed in 38 patients. There was no statistically significant association between the presence of a fistula and the treatment method or success rate (Table 4).

**Table 4:** The association between the treatment success and treatment methods and fistula formation

	Treatment success			
	No	Yes	Recurrence	*p*
Treatment methods				
Medical	9 (53%)	18 (41%)	0 (0%)	0.416
Surgery	0 (0%)	6 (14%)	0 (0%)	
Drainage	8 (47%)	20 (45%)	1 (100%)	
Fistula formation				
No	6 (35%)	17 (39%)	1 (100%)	0.435
Yes	11 (65%)	27 (61%)	0 (0%)	

*p:* Significance value for Chi-square test, *p < 0.05* was considered statistically significant

Seventeen patients who failed treatment were categorized as BI-RADS 3. Symptoms of 7 patients regressed spontaneously in the follow-up period. Also, one patient developed fistul and than recovered. The remaining 9 patients had pain and swelling. Sonography showed abscess in 5 patients and fistula tract in 1 patient. One of these patients underwent abscess drainage but treatment failed.

## Discussion

IGM is a recurrent, resistant chronic inflammatory disease, which shows great variety on MRI. Multiple heterogeneous areas, with ring-like enhanced micro-abscesses, regional NMEs or heterogeneous enhanced masses are the most common findings detected on MRI in cases of IGM [7]. Multiple micro-abscesses, ill defined enhanced masses, skin thickening, parenchymal distortion, oedema, fluid collection, nipple retraction, fistulas and axillary lymphadenopathy may also be seen on MRI [3]. In the present study, most of the patients had NMEs, in addition to abscesses and fistulas. These findings are in accordance with those in the literature [3, 7].

Due to the rarity of IGM and the great variety of its appearance on radiological imaging, the management and treatment of IGM are problematic. There are no standardized and optimal treatment options at present. A recent study on the potential role of sociodemographic factors in treatment failure in IGM reported that a history of pregnancy, breastfeeding, breast infections and smoking were risk factors for treatment failure [8]. The same study reported that current treatment methods did not affect IGM recurrence. Atak et al. found the highest potential for treatment failure in IGM patients with abscesses [9]. Sakurai et al. reported that patients who developed a fistula and an abscess were more difficult to treat [10]. In the present study, in contrast to the literature data, in the majority of patients, there was no association between treatment success and the presence of fistulas. Furthermore, the majority of patients with abscesses were treated successfully, with IGM recurrence in only one patient. As noted earlier, there is limited research on the association between radiological features and treatment failure. In the present study, according to the MRI findings, the affected quadrant was the only predictive factor in treatment success. Lesions located in the retroareolar region were more intractable to treatment, and treatment success was lower in such cases as compared with IGM involving other quadrants. This finding may be explained by the intensity of ductal inflammation in the retroareolar region. Although IGM is not thought to generally affect the retroareolar region, a recent study reported retroareolar involvement in a considerable number of patients with IGM [11]. In our study, retroareolar involvement was noted compatible with the findings of this recent study.

At present, the preferred treatment options for IGM are medical treatment [systemic steroids] or surgery. The medical treatment of IGM includes antibiotherapy, steroids and immunosuppressive drugs, such as methotrexate and azathioprine. The surgical treatment consists of abscess drainage, local and wide excision or a mastectomy. Although debate surrounds the most appropriate treatment method, some recent studies concluded that surgical methods were superior to medical methods [9, 12]. In the present study, in terms of the success rate, we found no statistically significant difference in the superiority of surgical versus medical treatment. Aggressive surgical interventions are not recommended as a first-line treatment modality for IGM because of poor cosmetic results. Patients with IGM should be managed on a case-by-case basis. According to a previous study, the addition of steroid therapy to surgical treatment can decrease the recurrence rate of IGM [13]. In the our study, only one patient was managed with steroid therapy, and no recurrence was observed in this patient during a 2-y follow-up. A previous study reported a recurrence rate of between 5 and 50% among all IGM patients, even when broad surgical excision was performed [14]. In the present study, IGM did not recur in any of the patients who received medical or surgical treatment, but it recurred in one patient who underwent drainage. We demonstrated that there was no relationship between MRI findings and the treatment success rate in IGM, except in cases of IGM involving the retroareolar region. The results explain the absence of a standardized treatment regimen for IGM and the difficulty in determining the optimum treatment.

The present study has some limitations. First, the data were collected retrospectively. Second, the sample size was relatively small.

## Conclusions

It is impossible to predict the treatment success or outcome using only MRI findings. However, prospective studies are needed to determine whether MRI findings of IGM can predict treatment success/failure and to improve treatment success of IGM. The treatment of IGM in the retroareolar region may be more failed compared to those in other quadrants.

## Data Availability

The datasets used and/or analysed during the current study available from the corresponding author on reasonable request.

## Abbreviations

BIRADS: Breast Imaging Reporting and Data System
IGM: Idiopathic granulomatous mastitis
MRI: Magnetic resonance imaging
NMEs: Non-mass enhancements
